# The Lectin ArtinM Induces Recruitment of Rat Mast Cells from the Bone Marrow to the Peritoneal Cavity

**DOI:** 10.1371/journal.pone.0009776

**Published:** 2010-03-22

**Authors:** Patricia Andressa de Almeida Buranello, Maria Raquel Isnard Moulin, Devandir Antonio Souza, Maria Célia Jamur, Maria Cristina Roque-Barreira, Constance Oliver

**Affiliations:** Department of Cell and Molecular Biology and Pathogenic Bioagents, Faculdade de Medicina de Ribeirão Preto, University of São Paulo, Ribeirão Preto, São Paulo, Brazil; Universidad Nacional, Costa Rica

## Abstract

**Background:**

The D-mannose binding lectin ArtinM is known to recruit neutrophils, to degranulate mast cells and may have potential therapeutic applications. However, the effect of ArtinM on mast cell recruitment has not been investigated.

**Methodology:**

Male Wistar rats were injected i.p. with ArtinM or ConA (control). The ability of the lectin to degranulate peritoneal and mesenteric mast cells was examined. Recruitment of mast cells to the peritoneal cavity and mesentery after ArtinM injection was examined with or without depletion of peritoneal mast cells by distilled water.

**Results:**

ArtinM degranulated both peritoneal and mesentery mast cells *in vitro*. Three days after i.p. injection of the lectin there were reduced numbers of mast cells in the peritoneal lavage, while at 7 days post injection of ArtinM, the number of peritoneal mast cells was close to control values. Since immature mast cells are recruited from the bone marrow, the effect of the lectin on bone marrow mast cells was examined. Injection of ArtinM resulted in an increased number of mast cells in the bone marrow. To determine if degranulation of mast cells in the peritoneal cavity was required for the increase in bone marrow mast cells, the peritoneal cavity was depleted of mast cells with ultrapure water. Exposure to ArtinM increased the number of mast cells in the bone marrow of rats depleted of peritoneal mast cells.

**Conclusions:**

The ArtinM induced recruitment of mast cells from the bone marrow to the peritoneal cavity may partially explain the therapeutic actions of ArtinM.

## Introduction

The D-mannose binding lectin, ArtinM, previously called KM+ or Artocarpin, is known modulate the immune response, activating macrophages [1], neutrophils [2] and mast cells [3]. Previous studies have shown that in mice, administration of ArtinM stimulates IL-12p40 production by macrophages and dendritic cells [1]. As a consequence ArtinM induces Th1 protective responses against *Leishmania major* [1], *Leishmania amazonensis* [4] and *Paracoccidioides brasilensis* [5]. Recently, topical treatment with ArtinM has been shown to accelerate corneal epithelial wound healing in rabbits [6]. Thus, ArtinM may have potential therapeutic applications.

ArtinM is known to activate mast cells either by binding IgE bound to the high affinity IgE receptor (FcεRI) or by binding directly to FcεRI [3]. Activation of mast cells via FcεRI causes the immediate release of pre-formed mediators, the production of newly formed lipid mediators and 8 to 12 hours after activation the release of newly synthesized cytokines and growth factors [7], [8]. Following i.p. injection of ArtinM in rats, there is recruitment of neutrophils to the peritoneal cavity [9]. Further studies indicated that mast cell activation provides an amplification loop for the ArtinM induced neutrophil recruitment [3]. However, in these studies, the effect of ArtinM on mast cell recruitment was not investigated.

Mast cells play a role in many physiological and pathological processes [10] including allergy, inflammation, cancer, and heart disease, [11], [7] and are also important in maintaining tissue homeostasis [12]. They are a major source of cytokines and chemokines giving them importance as immunoregulatory cells. Mast cells are also critical cellular components in such processes as wound healing [13]. At the early stages of wound healing, mast cells liberate various mediators that recruit other cell types to the site of injury. At the later stages, mast cells themselves are recruited to the wound site and contribute to wound maturation and remodeling [14], [15].

Because of the potential pharmacological applications of ArtinM, the present study was undertaken to examine the effect of ArtinM on mast cell degranulation and recruitment *in vivo* in rats. The ability of ArtinM to degranulate mast cells in the peritoneal cavity and mesentery and the recruitment of mast cells from the bone marrow to the peritoneal cavity were examined. The results demonstrate that ArtinM is highly efficient in recruiting mast cells from the bone marrow to the peritoneal cavity and indicate the value of ArtinM as an immunomodulatory agent.

## Methods

### Ethics Statement

The research was conducted in accordance with “Ethical principles in the use of experimental animals” adopted by the Brazilian College of Animal Experimentation. Experimental protocols were approved by the Commission on Ethics on Animal Experimentation of the Faculdade de Medicine de Ribeirão Preto (Protocol numbers 019/2005 and 152/2005). Animals were anesthetized with ketamine 80 mg/kg plus xylazine 12 mg/kg (Sigma-Aldrich, St. Louis, MO) prior to experimental treatment.

### Purification of ArtinM

The lectin ArtinM was purified by affinity chromatography as previously described [9]. The purity of the preparation was analyzed by sodium dodecyl sulfate-polyacrylamide gel electrophoresis (SDS-PAGE) and the protein concentration determined by measuring the absorbance at 280 nm.

### Animals

Male Wistar rats, weighing 150 g, were obtained from the Central Animal Facility of the Faculdade de Medicina de Ribeirão Preto. All experiments were conducted according to the guidelines of the Faculdade de Medicina de Ribeirão Preto (Protocol numbers 019/2005 and 152/2005). Animals were anesthetized with ketamine 80 mg/kg plus xylazine 12 mg/kg (Sigma-Aldrich, St. Louis, MO) prior to experimental treatment. All experiments were done in triplicate.

### Cells

Peritoneal cells were obtained by injecting rats i.p. with 15 ml PBS (phosphate buffered saline). The peritoneal washing was collected with a Pasteur pipette after laparotomy and the cells washed by centrifugation (x201*g*) in PBS prior to use. The cells were placed on coverslips coated with Biobond (Electron Microscopy Sciences, Hatfield, PA) fixed in 2% formaldehyde (EM Sciences) and stained for 15 min in 0.1% Toluidine blue-1% acetic acid, pH 2.8.

Bone marrow cells were obtained by flushing the marrow cavity with PBS containing 2% BSA (Sigma-Aldrich), 1000 U/ml deoxyribonuclease I (Sigma-Aldrich) and 1000 U/ml heparin (Produtos Roche Químicos e Farmacêuticos S.A., Rio de Janeiro, R.J.). The marrow was dissociated by aspirating the cells with a Pasteur pipette and passing the suspension through a 25 µm nylon filter. The cells were washed by centrifugation (x201*g*) in PBS prior to use.

### Mesentery

The mesentery was removed from animals along with the intestines and placed in Petri dishes whose bottoms were covered with paraffin. The mesenteries were stretched over the paraffin and secured with fine needles. The mesentery was then fixed in 10% formalin (J.T. Merck, Darmstadt, Germany) in PBS and stained for 15 min in 0.1% Toluidine blue-1% acetic acid, pH 2.8. Mesentery fragments were stretched over glass slides, dried on a heating plate at 45°C, dehydrated in a graded series of ethanol (Merck), cleared in xylene (Merck), and coverslips mounted with Permount (Fisher Scientific, Atlanta, GA).

### Treatments

#### Lectins

For experiments *in vivo*, animals were injected i.p. with 4 ml of PBS containing either 10 µg or 200 µg ArtinM or ConA (Sigma Aldrich, St. Louis, MO). For *in vitro* experiments the peritoneal lavage or mesentery fragments were incubated in 1 ml Iscove's Modified Dulbecco's Medium (Sigma-Aldrich) containing 10 µg or 200 µg of ArtinM or ConA at 30°C for 1 h. The lectin concentrations chosen were based on data in the literature [3], [16] as well as on preliminary dose-response experiments. For some experiments, the lectins were preincubated with D-galactose (25 mM; Sigma-Aldrich), D-glucose (25 mM; Sigma-Aldrich), D-mannose (25 mM; Sigma-Aldrich) or mannotriose (Manα1-3[Manα1-6]Man (10 mM; Dextra Laboratories, Reading, UK). Control animals were injected i.p. with 4 ml PBS.

#### Mast cell depletion of the peritoneal cavity

Mast cell depletion of the peritoneal cavity by distilled (ultrapure) water is a widely used method [17]–[19]. Although all cells types in the peritoneal cavity are exposed to distilled water, only the mast cells, because of their granule composition, are lysed by distilled water [20]. In order to deplete the peritoneal cavity of mast cells, animals were injected with 15 ml i.p. of ultrapure, sterile water (Milli-Q; Millipore, Billerica, MA).

#### Mast cell degranulation

Animals were injected i.p. with 200 µg of Compound 48/80 (Sigma-Aldrich) (50 µg/ml) in 4 ml of 0.02 M Tyrode-Tris buffer, pH 7.2 [21].

#### Repopulation of the mesentery

Repopulation of the mesentery by mast cells was examined in animals with or without depletion of the peritoneal mast cells. In animals not depleted of peritoneal mast cells, 10 µg or 200 µg of ConA in 4 ml PBS was injected i.p. Seven days later, the mesentery was removed, fixed in 2% formaldehyde (EM Sciences), and stained for 15 min in 0.1% Toluidine blue-1% acetic acid, pH 2.8. Control animals received only PBS. Animals depleted of mast cells by ultrapure water received 10 µg ConA or 10 µg ArtinM in 4 ml PBS i.p. 24 h after injection of the water. Control animals received only ultrapure water. Seven days after injection of the lectins, the mesentery was removed, fixed in 2% formaldehyde (EM Sciences), and stained for 15 min in 0.1% Toluidine blue-1% acetic acid, pH 2.8.

#### *In vitro* degranulation assays

For *in vitro* degranulation assays, prior to fixation, mesentery fragments were incubated with 10 µg/ml of either lectin. Controls were incubated in medium without lectin. After incubation, the fragments were rapidly washed in PBS, fixed in 2% formaldehyde (EM Sciences) and, stained for 15 min in 0.1% Toluidine blue-1% acetic acid, pH 2.8.

### Quantification of peritoneal and mesenteric mast cells

Images were acquired by bright field microscopy with a 40X objective using a Nikon Eclipse E800 microscope (Nikon USA, Melville, NY) equipped with a Nikon DXM1200 digital camera. Images from 10 (peritoneal lavage) or 16 (mesentery) separate fields from 3 separate rats for each experimental condition were acquired. All fields of the mesentery included a mesenteric blood vessel. For the peritoneal lavage, the number of granulated and degranulated mast cells in each field was determined. For the mesentery the number of mature and immature mast cells, based on degree of metachromasia, was analyzed.

### Immunomagnetic isolation of mast cells

Tosyl activated Dynabeads (Invitrogen, Dynal Biotech, Carlsbad, CA) were conjugated with mAb AA4, that recognizes unique gangliosides on the surface of granulated rodent mast cells [22], [23], or with normal mouse IgG (Jackson ImmunoResearch, West Grove, PA) as previously described [24]. Briefly, mAb AA4 (300 µg/ml) was dissolved in 0.05 M borate buffer, pH 9.5 and mixed with tosylactivated Dynabeads. The solution of mAb AA4 was incubated for 24 h at 22°C with slow end-over-end rotation. After incubation the Dynabeads were collected with a magnetic particle concentrator (MPC; Dynal). The coated beads were then washed, using the MPC, and collected with the MPC, the supernatant discarded, and the beads resuspended in PBS containing 0.1% BSA to a concentration of 30 mg/ml and stored at 4°C until use. Normal mouse IgG was coupled to tosylactivated Dynabeads using the protocol for cell separation applications as recommended by the manufacturer.

Mast cells were immunomagnetically isolated from the peritoneal lavage and from the bone marrow using mAb AA4 coupled to tosylactivated Dynabeads M-450 as previously described [25], [26]. After isolation, the cells were rinsed in PBS, fixed in 2% formaldehyde (EM Sciences), and stained for 15 min in 0.1% Toluidine blue-1% acetic acid, pH 2.8.

The percentage of mast cells was determined by counting the cells using a Neubauer Camera. Cells attached to the magnetic beads (positive population) as well as the negative population were counted.

### Infusion of CellTracker labeled cells

After immunomagnetic isolation, bone-marrow derived mast cells were incubated for 30 min at 37°C in Iscove's Modified Dulbecco's Medium without serum containing *CellTracker* Red CMTPX (Invitrogen, Molecular Probes, Carlsbad, CA). After labeling, the cells were incubated for an additional 30 min at 37°C in Iscove's Modified Dulbecco's Medium with 10% heat inactivated fetal bovine serum (Invitrogen, GIBCO, Carlsbad, CA). Cell labeling was monitored by fluorescence microscopy using a Nikon Eclipse E800 fluorescent microscope.

Before the infusion of *CellTracker* labeled cells, the peritoneal cavity was depleted of mast cells by i.p. injection of 15 ml of purified water. Then, 2.5×10^5^ cells/rat, labeled with *CellTracker* Red CMTPX in 500 µl Iscove's Modified Dulbecco's Medium were injected via the caudal vein. Control animals were injected with only 500 µl Iscove's Modified Dulbecco's Medium. Two hours after cell infusion, some animals received i.p. 10 µg ArtinM in 4 ml PBS. Control animals received only PBS. At 24 h and 48 h after cell infusion, cells were collected from the peritoneal cavity by laparotomy using a Pasteur pipette after injecting 15 ml PBS into the cavity. Cells were also collected from the bone marrow as described above. The cells were rinsed by centrifugation 5 times in PBS, fixed with 1% formaldehyde (Electron Microscopy Sciences, Hatfield, PA) and analyzed by flow cytometry (BD FACSCalibur™, BD Biosciences, Franklin Lakes, NJ).

### Statistical Analysis

The experimental results were analyzed using non paired Student's t-test. Differences were considered significant at *p≤0.05 and **p≤0.01.

## Results

In the present study the ability of ArtinM to recruit mast cells from the bone marrow to the peritoneal cavity of rats was examined. In order to determine if mast cell recruitment was due solely to lectin induced degranulation of peritoneal mast cells, the effect of ArtinM was also examined in a model system in which the peritoneal cavity was depleted of mast cells prior to administration of the lectin. The specificity of the effects of ArtinM was analyzed by comparing the results with ArtinM to those with ConA. ConA, a glucose/mannose binding lectin, is well characterized for its ability to degranulate mast cells [8], [27]–[29].

### The lectin ArtinM degranulates peritoneal and mesenteric mast cells

**Figure 1:**
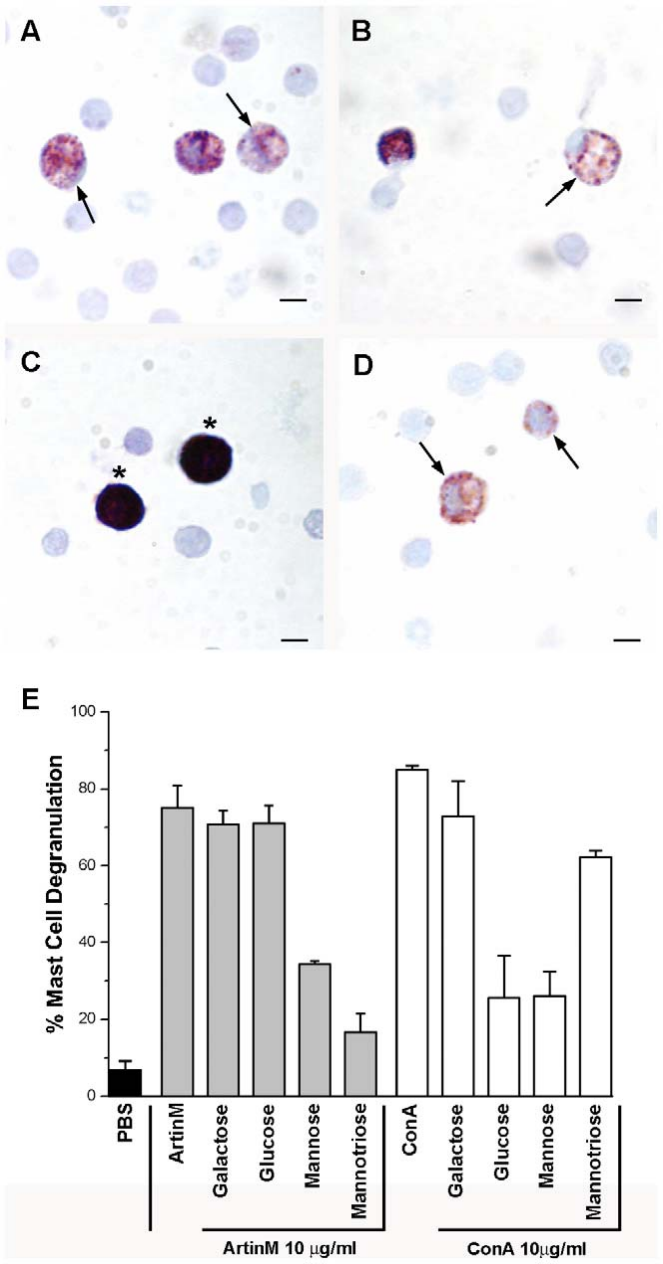
ArtinM and ConA degranulate peritoneal mast cells *in vitro* in a lectin specific manner. The total cell population from the peritoneal lavage was incubated with 10 µg of ArtinM (A) or ConA (B). Control cells were incubated in PBS (C). Compound 48/80 (D) was used as a positive control for degranulation. Stained with Toluidine blue. (Arrows, degranulated mast cells; Asterisks, intact mast cells) Bars = 10 µm. Quantification of peritoneal mast cell degranulation (E). The data shown is the average±SD of 3 separate experiments.

In order to examine the effect of the lectin ArtinM on mast cell degranulation, the total cell population from the peritoneal lavage was incubated with ArtinM or ConA. After staining with toluidine blue, the preparation was examined by bright field microscopy and the percent of degranulated mast cells was determined. Incubation with 10 µg/ml of either ArtinM or ConA (Fig. 1A and B) resulted in degranulation of 75%±5.7% and 85%±1.1%, respectively, of peritoneal mast cells *in vitro*. Incubation with PBS did not elicit degranulation (Fig. 1C) and incubation with Compound 48/80 resulted in virtually 100% degranulation (Fig. 1D). In order to determine if this degranulation was dependent on the carbohydrate recognition domain (CRD) of the lectins, the lectins were assayed following preincubation with various sugars. Mast cell degranulation was inhibited only by the lectin specific sugars (Fig. 1E). When ArtinM was preincubated with 25 mM D-mannose or 10 mM mannotriose, only 34.4±0.9% and 16.7±4.8% respectively of the mast cells were degranulated. When ConA was preincubated with 25 mM D-glucose or 25 mM D-mannose only 25.6±10.9% and 26.1±6.4% respectively of the mast cells were degranulated.

**Figure 2:**
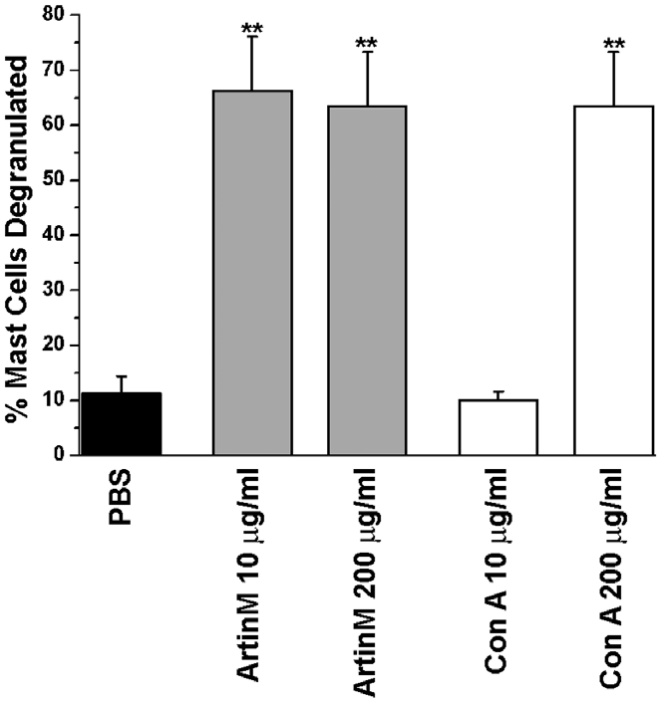
ArtinM and Con A also degranulate mast cells in vivo. The peritoneal lavage was examined 1 hr after i.p. injection of 10 µg of either lectin. There was no significant difference in the ability the two lectins to degranulate mast cells. **p≤0.01. The data shown is the average±SD of 3 separate experiments.

The effect of ArtinM and ConA on peritoneal mast cell degranulation *in vivo* was examined by injecting rats i.p. with either lectin. At 10 µg/rat only ArtinM resulted in mast cell degranulation (Fig. 2). However at a higher concentration (200 µg/rat), both lectins degranulated peritoneal mast cells to the same extent. These results demonstrate that the lectins ConA and ArtinM have the ability to degranulate peritoneal mast cells *in vivo* as well as *in vitro*.

**Figure 3:**
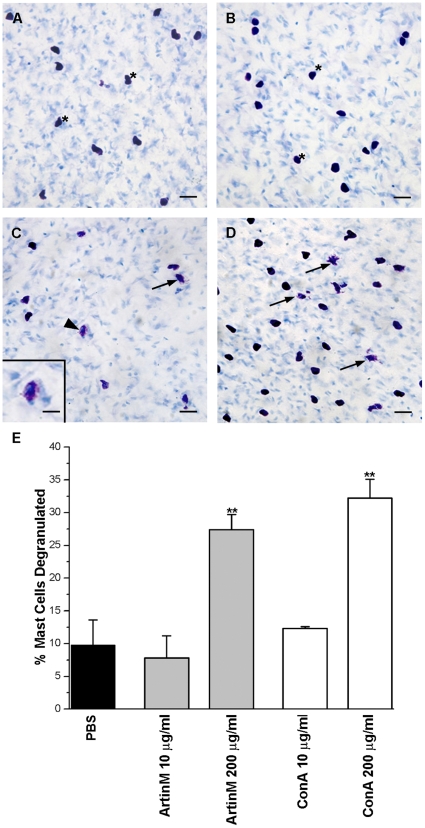
ArtinM and ConA also degranulate mesenteric mast cells *in vitro* only at high concentrations. Mesenteries were incubated with 10 µg/ml ArtinM (A), 10 µg/ml ConA (B), 200 µg/ml ArtinM (C) or 200 µg/ml ConA (D). (Arrows, degranulated mast cells; *, non degranulated mast cells). Bar = 20 µm. Inset (C): Details of degranulated mast cell indicated by arrowhead. Bar = 10 µm Stain: Toluidine blue. Quantification of mesenteric mast cell degranulation by ArtinM or ConA (E). At 200 µg of either ArtinM or ConA there was significant (**p≤0.01) degranulation of mesenteric mast cells. The data shown is the average±SD of 3 separate experiments.

In order to determine if the lectins ArtinM and ConA could also degranulate mesenteric mast cells, mesenteric fragments were incubated with 10 µg/ml of each lectin. Neither lectin induced mast cell degranulation (Fig. 3A and B) at this concentration. However, when the mesenteric fragments were incubated with the lectins at 200 µg/ml mast cell degranulation was observed (Fig. 3C and D). The percent of cells degranulated, 27.4%±2.3% of the mesenteric mast cells incubated with ArtinM and 32.2%±2.9% of the mesenteric mast cells incubated with ConA, was significantly greater than mesenteric mast cells incubated only with PBS (Fig. 3E). Although both lectins have the ability to degranulate mast cells, higher concentrations of the lectins are needed to degranulate mast cells in the mesentery. This is presumably due to the fact that the lectins also bind to the extracellular matrix surrounding the mesenteric mast cells thus reducing the amount of lectin available to bind to the mast cells.

**Figure 4:**
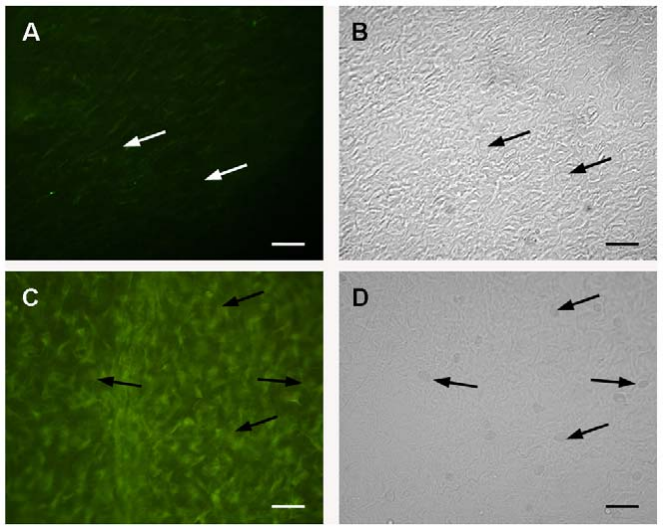
Con A-FITC binds preferentially to the extracellular matrix. Rats were injected i.p. with 10 µg Con A-FITC (A. Fluorescence image, B. DIC image) or with 200 µg Con A-FITC. (C. Fluorescence image, D. DIC image). (Mast cells, arrows).

The ability to degranulate mesenteric mast cells *in vivo* appeared to be dose dependent. In order to determine if this dose dependency is related to the binding of lectin to the extracellular matrix, animals were injected with 10 µg/ml or 200 µg/ml (4 ml/animal) of Con A conjugated to FITC. At a concentration of 10 µg/ml of Con A there was only a faint labeling of the extracellular matrix and none of the mast cells were labeled (Fig. 4A and B.). However, after IP injection of 4 ml of 200 µg/ml of Con A conjugated to FITC, the lectin bound strongly to the fibers in the extracelular matrix and mast cells were labeled (Fig. 4C and D). These results indicate that *in vivo* at low concentrations there is not sufficient lectin available to bind directly to the mast cells. It is also possible that at high concentrations, the lectin persists in the peritoneal cavity for a longer time.

### Intraperitoneal injection of ArtinM and ConA results in a reduction in the number of metachromatic peritoneal mast cells

**Figure 5:**
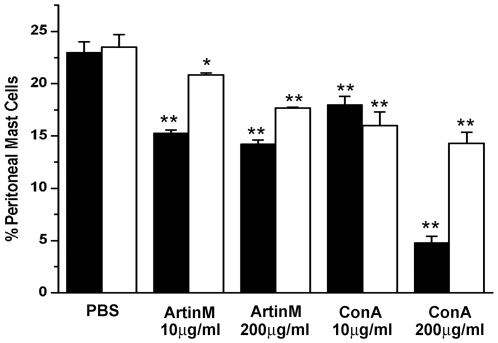
Intraperitoneal injection of ArtinM and ConA reduces the percentage of metachromatic mast cells in the peritoneal lavage. Animals were injected i.p. with 4 ml of PBS containing either 10 µg or 200 µg of ArtinM or ConA, and the percent of metachromatic mast cells present in the peritoneal lavage was examined at 3 and 7 days post injection. The results shown are the average±SD of 3 separate experiments. 3 days post injection: ▪; 7 days post injection: □. *p≤0.05, **p≤0.01.

The number of metachromatic mast cells was examined in the peritoneal lavage at 3 and 7 days after i.p. injection of the lectins. At 3 days post stimulation, released granule matrix has been cleared from the peritoneal cavity and secretory granule resynthesis is complete [21]. When the peritoneal lavage was examined 3 days after i.p. injection of 10 µg ArtinM, there was a 32%±0.3% reduction in the number of metachromatic mast cells. By 7 days the percent of metachromatic peritoneal mast cells was almost the same as that seen in the control animals (Fig. 5). IP injection of 200 µg of ArtinM was no more effective in reducing the mast cell number than 10 µg ArtinM. In contrast, after i.p. injection of 10 µg ConA, the number of metachromatic peritoneal mast cells had diminished by 21.8%±0.8% and by 7 days was reduced by 32%±1.3% (Fig. 5). However, when animals were injected with 200 µg/ml of ConA at 3 days, the percent of metachromatic mast cell dropped by 80%±0.6% in comparison with the control cells, and by day 7 the percent of mast cells was reduced by 40%±1.1% of the control values. High concentrations of ConA had a more pronounced effect on the mast cell numbers than increased concentrations of ArtinM. The reduction in the number of metachromatic peritoneal mast cells seen at 3 and 7 days post injection, most likely represents a combination of the direct effect of the lectins on mast cells and the indirect effects mediated by the action of ArtinM on other cells, such as neutrophils [2].

### IP injection of ConA, but not ArtinM results in an increased number of immature mast cells in the mesentery

**Figure 6:**
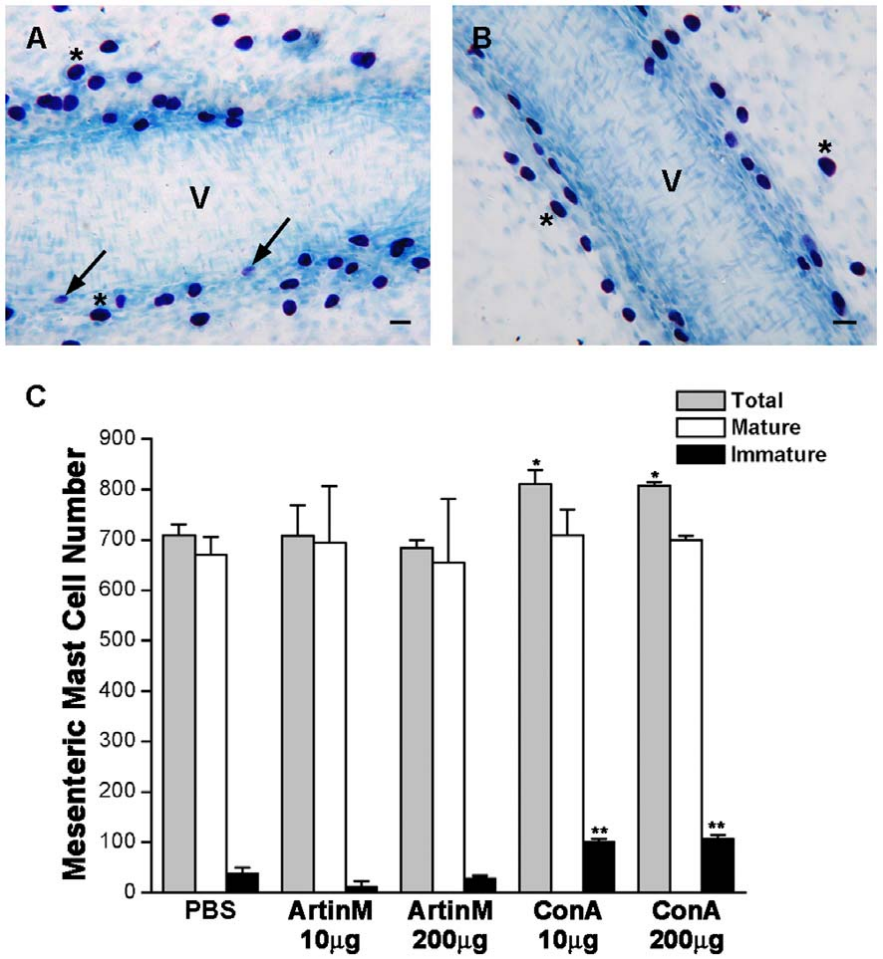
ConA, but not ArtinM recruits immature mast cells to the mesentery. In the mesentery, 7 days after i.p. injection of 200 µg of Con A immature mast cells (arrows) with few metachromatic granules as well as mature mast cells (*) were found next to a blood vessel (A). In animals injected with PBS, only mature mast cells (*) were seen along blood vessels (B). Stain: Toluidine blue. (C) Seven days after i.p. injection of 10 µg or 200 µg of ArtinM or ConA the total number of mesenteric mast cells as well as the number of mature and immature mast cells was determined. *p≤0.05. The results shown are the average±SD of 3 separate experiments.

Seven days after injection of the lectins, when mature and immature mast cells can be identified by their degree of metachromasia, the mesenteries from animals injected with either 10 µg or 200 µg of ArtinM were examined. The total mast cell number was similar to that seen in non injected animals and the numbers of mature and immature mast cells were unaltered (Fig. 6C). In contrast, animals injected with 10 µg or 200 µg ConA showed an increase in the total number of mast cells in the mesentery (Fig. 6C). Based on the degree of metachromasia, following injection of ConA, the number of immature mast cells seen in the mesentery, especially along blood vessels (Fig. 6A) was 3 times the number seen in the PBS injected animals (Fig. 6B). However, the number of mature mast cells was unaltered. These results suggest that in the presence of ConA, mast cell degranulation may not be necessary for recruitment of immature mast cells and that ConA may be acting as a chemotactic factor.

### IP injection of ArtinM or ConA results in an increase in the number of mast cells in the bone marrow

**Figure 7:**
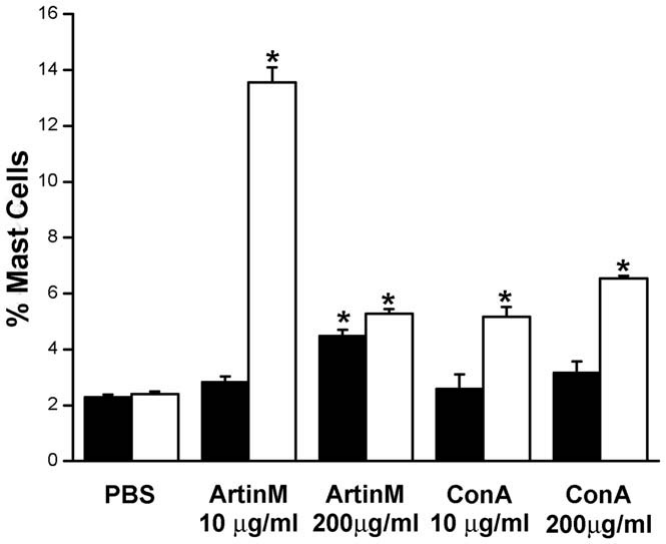
Intraperitoneal injection of lectin increases the percent of mast cells in the bone marrow. Animals were injected i.p. with Artin M or ConA. At 3 (▪) or 7 (□) days after injection mast cells were immunomagnetically isolated from the bone marrow using mAb AA4 and the percent of mast cells determined. *p≤0.05. The results shown are the average±SD of 3 separate experiments.

Since repopulation of mast cells at peripheral sites is thought to occur by recruitment of bone marrow mast cells [26], [30], it was of interest to investigate the changes in the number of bone marrow mast cells (Figure 7). Mast cells were immunomagnetically isolated from the bone marrow using mAb AA4 conjugated to magnetic beads. mAb AA4 recognizes mast cell specific gangliosides on the surface of very immature, immature and mature rodent mast cells (granulated mast cells). Three days after i.p. injection of 10 µg of ArtinM there was no difference in the number of bone marrow mast cells as compared to animals injected with only PBS. However, by 7 days after injection there was a 456%±0.6% increase in mast cells in the bone marrow. With 200 µg of ArtinM at 3 days there was an increase of 102±0.2% in bone marrow mast cells and by 7 days the number had increased to 124%±0.2% (Fig. 7). The same experiments were repeated using ConA. Although at 3 days after i.p. injection of 10 µg ConA, there was no significant change in the percent of mast cells present in the bone marrow, by 7 days there was a 115%±0.4% increase in the mast cells in comparison to PBS injected controls. In contrast, 3 days after i.p. injection of 200 µg ConA there was a 38%±0.4% increase in the percent of bone marrow mast cells and after 7 days the percent had increased to 172%±0.1% in comparison to controls. Therefore, at 7 days post injection of either ArtinM or ConA the percentage of mast cells in the bone marrow was dramatically increased. Furthermore, 10 µg ArtinM was most effective in increasing the mast cell population in the bone marrow. These data suggest that the effects of i.p. lectin injection extend beyond the peritoneal cavity and also affect mast cells in the bone marrow.

### ArtinM is efficient in recruiting mast cells to the peritoneal cavity and to the mesentery in animals depleted of peritoneal mast cells

**Figure 8:**
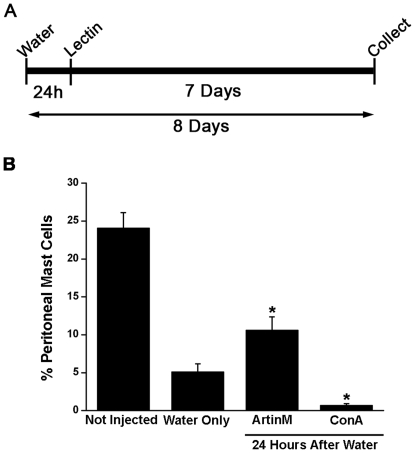
ArtinM recruits mast cells to the peritoneal cavity in mast cell depleted animals. Animals were injected i.p. with purified water to deplete the peritoneal cavity of mast cells and 24 h later received an i.p. injection of 4 ml of PBS containing 10 µg of ArtinM or ConA. The peritoneal lavage was collected 7 days after lectin injection (A). The percent of peritoneal mast cells was determined under the various conditions (B). *p≤0.05. The results shown are the average±SD of 3 separate experiments.

In order to investigate the contribution of peritoneal mast cells in the recruitment of mast cells following ArtinM, animals were injected i.p. with ultrapure water to deplete the peritoneal cavity and the mesentery of mast cells [17], [20]. Twenty-four hours later, animals were injected i.p. with ArtinM or ConA (10 µg/rat) and 7 days after lectin injection, the peritoneal lavage and mesentery were collected and the mast cells quantified (Fig. 8A). At this time repopulation of the peritoneal cavity is underway and immature and mature mast cells can be identified by their metachromasia [18]. Under these conditions, ArtinM is effective in recruiting mast cells to the peritoneal cavity (Fig. 8B). In comparison to the non-depleted animals, which had 24.1% mast cells in the peritoneal lavage, animals injected with water only had 5.1%±1.0% mast cells in the peritoneal lavage. In contrast, animals injected with ArtinM had twice the percentage of peritoneal mast cells in comparison with animals injected only with water and 15 times the percentage of mast cells in comparison with animals injected with ConA. In animals injected with ConA, the percentage of peritoneal mast cells was significantly lower (0.7%±0.2%) in comparison to the animals injected only with water or with ArtinM.

**Figure 9:**
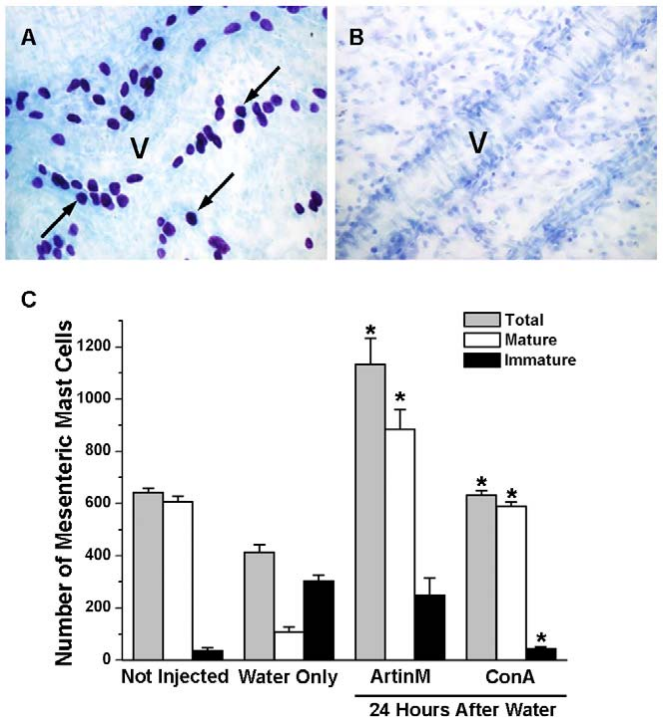
Intraperitoneal lectin injection recruits mast cells to the mesentery in mast cell depleted animals. Animals were injected i.p. with purified water to deplete the peritoneal cavity of mast cells and 24 h later received an i.p. injection containing 10 µg of ArtinM or ConA. The mesentery was collected 7 days after lectin injection. In uninjected animals (A) the mesentery was replete with mature mast cells. Many lying adjacent to blood vessels (V). Twenty four hours after injection of purified water (B) no mast cells could be seen in the mesentery. The total number of mesenteric mast cells as well as the number of mature and immature mesenteric mast cells was determined under the various conditions (C). *p≤0.05. The results shown are the average±SD of 3 separate experiments.

Intraperitoneal injection of with ultrapure water is also efficient in depleting the mesentery of mast cells (Fig. 9) [17], [20]. Twenty four hours after i.p. injection of purified water, no mast cells were present in the mesentery (Fig. 9B), but by 8 days after water injection, the total number of mast cells was returning to normal (Fig. 9C). However in comparison to non depleted animals, the water injected animals had significantly more immature mast cells than mature mast cells. In contrast, following i.p. injection of ArtinM into depleted animals the total number of mast cell was 1.8 times that seen in non depleted animals and 2.2 times greater than in the animals that received water only. This difference was largely due to increased numbers (883.5±75.7) of mature mast cells and there was no significant difference between the number of immature mast cells seen in depleted animals injected with ArtinM or with water only. Injection of ConA, i.p., resulted in an 1.5 times increase in the total number of mast cells as compared with animals injected with water only. This increase in total mast cell number was due to a preferential increase in the number (588.5±16.3) of mature mast cells, in relation to the number (109±18.4) of mature mast cells seen in animals injected only with water. This was accompanied by a reduction in the number (44.5±7.8) of immature mast cells. In the absence of mast cells in the peritoneal cavity, both lectins were efficient in recruiting mast cells to the peritoneal cavity and the mesentery, although ArtinM was considerably more effective. In addition, the lectins, especially ArtinM, appeared to accelerate mast cell maturation. These data further indicate that degranulation of peritoneal mast cells by the lectins is not essential for mast cell recruitment.

### IP injection of ArtinM results in an increase in the number of mast cells in the bone marrow of rats depleted of peritoneal mast cells

**Figure 10:**
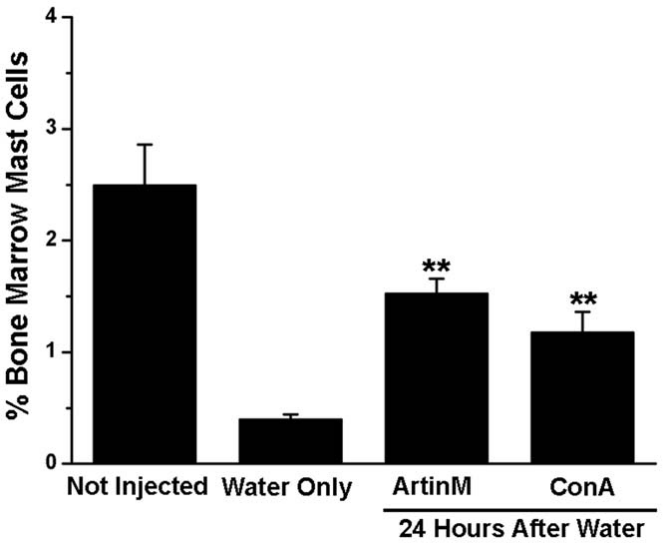
Intraperitoneal Injection of either ArtinM or ConA resulted in a significant increase in the percent of mast cells in the bone marrow. Eight days after injection of water and 7 days after injection of 10 µg of ArtinM or ConA, mast cells were immunomagnetically isolated from the bone marrow with mAb AA4 conjugated magnetic beads. **p≤0,01. The results shown are the average±SD of 3 separate experiments.

Since mast cells were recruited to the peritoneal cavity following injection of ArtinM or ConA, the effect of lectin administration on mast cell numbers in the bone marrow following mast cell depletion in the peritoneal cavity was also assessed. Animals were injected i.p. with ultrapure water. Twenty-four hours later, animals were injected i.p. with ArtinM or ConA (10 µg/rat) and 7 days after lectin injection, mast cells were isolated immunomagnetically from the bone marrow using mAb AA4 (Fig. 10). In the non-depleted animals 2.5±0.4% of the bone marrow cells were mast cells. At 8 days after ultrapure water, all of the depleted animals had significantly reduced numbers of mast cells in the bone marrow in comparison to the non-depleted controls. In animals that received only water the percent of mast cells was reduced to 0.4%±0.04%. However, the depleted animals that had been injected with ArtinM had 3 times as many mast cells (1.53±0.1%) as the depleted animals that had not received the lectin and in the animals injected with ConA the percentage was only 1.18±0.2% (Fig. 10). These results indicate that the lectins may have an indirect effect by increasing the number of mast cells in the bone marrow, thus providing a source of mast cells for repopulation of the peritoneal cavity.

### ArtinM augmented the homing of infused bone marrow derived mast cells to the peritoneal cavity

**Figure 11:**
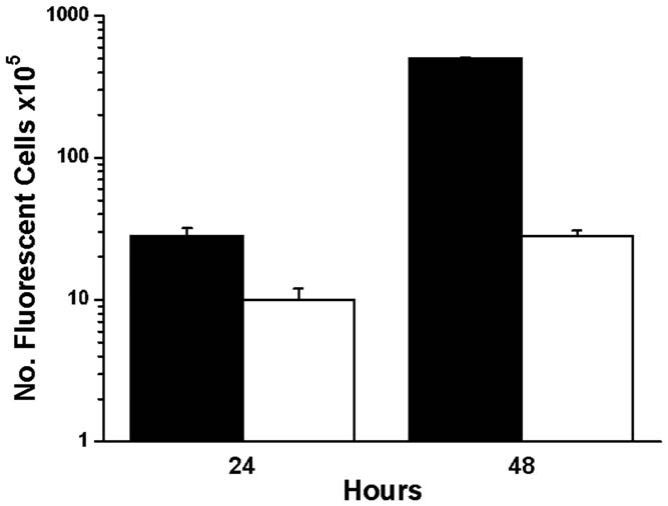
ArtinM increases the number of bone marrow derived mast cells recruited to the peritoneal cavity. The peritoneal cavity was depleted of mast cells by injection of ultrapure water prior to the infusion of immunomagnetically isolated bone marrow derived mast cells labeled with *CellTracker Red CMTPX*™ and i.p. injection of ArtinM. At 24 and 48 h after infusion, the presence of fluorescently labeled mast cells in the peritoneal lavage was analysed by FACS. Each data point represents the average number of cells±S.D. from 3 separate experiments. 100,000 cells were counted per time point in each experiment. (▪ with ArtinM; □ without ArtinM).

In order to further investigate if bone marrow mast cells are involved in the ArtinM induced mast cell repopulation of the peritoneal cavity, mast cells were isolated from the bone marrow, labeled with *CellTracker*™, and infused into animals whose peritoneal cavities had been depleted of mast cells (Fig. 11). In control animals a small number of these bone marrow derived mast cells homed to the peritoneal cavity. Injection of ArtinM, i.p., increased the homing 18 fold. These results further confirm the role of ArtinM as a chemoattractant for mast cells.

## Discussion

The present study demonstrates that following i.p. injection of the lectins ArtinM and ConA mast cells are recruited from the bone marrow to the peritoneal cavity and mesentery in rats, indicating that local administration of lectin may have consequences at distant sites. I.p. injection of the lectins also results in increased numbers of mast cells in the bone marrow that could be serving as a source of mast cells in order to repopulate the peritoneal cavity. This investigation further shows that ArtinM was more efficient than ConA in recruiting mast cells to the peritoneal cavity and more effective in increasing the number of mast cells in the bone marrow, suggesting that the overall response may be specific to a given lectin.

Exposure to ArtinM and ConA results in peritoneal mast cell degranulation at both low (10 µg) and high doses (200 µg) while mesentery mast cells degranulate only at high concentrations. The higher doses of lectin required for degranulation of mesenteric mast cells may be due to the fact that the lectins bind to the extracellular matrix surrounding the mast cells, thus lowering their effective concentration. The results of the present study confirm previous observations concerning mast cell degranulation induced by ArtinM [3], ConA [27], [30], [31] and by other mannose/glucose-binding lectins [32]. They are also similar to the results seen with lectins that have specificities other than mannose and mannose/glucose, such as the GalNAc-binding lectin from *Dolichos biflorus* [33]and the chitin-binding lectin from *Solanum tuberosus* [34]. However, the mast cell degranulation induced by these lectins is attributed to its binding to oligosaccharides linked to IgE, whereas ArtinM causes degranulation of RBL-2H3 cells whether or not the cells have IgE bound to FcεRI [3], suggesting that ArtinM may be interacting with mannose containing glycans on FcεRI itself. Analysis of the structure and composition of FcεRI in RBL-2H3 cells found that carbohydrates comprise 32% of the receptor [35]. Among the sugars present are galactose, manose, glycosamine and fucose. The extracellular domain of the α subunit in humans, that binds IgE, has 7 possible N-glycosylation sites while the α subunit of FcεRI in mice and rats has 6 and 7 potential N-glycosylation sites respectively. Thus, it is probable that contiguous IgE receptors are cross-linked by tetrameric ArtinM *in vivo*.

The increased numbers of mast cells in the bone marrow 7 days after treatment with ArtinM and to a lesser extent with ConA indicate that i.p. injection of these lectins is stimulating mast cell proliferation in the bone marrow. The bone marrow serves as a reservoir of immature mast cells that can be recruited to peripheral sites [25], [26]. This was confirmed in the present study by the increase in bone marrow derived mast cells that homed to the peritoneal cavity following ArtinM administration. Interaction between soluble or membrane bound lectins and cells from the immune system can activate these cells [36]–[38]. Among the processes that may be stimulated by exposure to lectins is cell proliferation. Although little is known about the ability of lectins to induce mast cell proliferation, a wide variety of lectins have been shown to be mitogenic for various other cell types [39], [40]. Therefore, it would be reasonable to expect that ArtinM would also stimulate mast cell proliferation. Preliminary studies in our laboratory have shown that ArtinM can stimulate mast cell proliferation at concentrations below those needed for degranulation. This fact may also explain the ability of ArtinM to act as a therapeutic agent [4], [6] without eliciting an allergic response.

The migration of mast cells is important in many different processes such as inflammation, allergy, tissue repair and tumorigenesis [30], [41]. The ability of any plant lectin to recruit mast cells has not been reported. In this study, we showed that ArtinM and ConA were efficient in recruiting mast cells from the bone marrow after i.p injection of the lectins in non-depleted animals. In analogy with leukocyte recruitment this suggests that soluble chemoattractants may act jointly with adhesion molecules to regulate the homing of immature mast cells to tissues [16]. These chemoattractants may also be involved in the release of progenitors from the bone marrow. Plant lectins that bind to mannose or galactose may interact with various endogenous molecules involved in innate and acquired immune responses. There are several published studies indicating that some plant lectins may mimic endogenous lectins in animals and induce migration of neutrophils and mononuclear cells *in vitro* and *in vivo* [9], [42]. During cell migration, the cells interact with their surrounding environment by means of cell-cell interactions or cell-matrix interactions [43]–[47]. Mast cell migration induced by ArtinM may occur by a process similar to that reported for neutrophils. Santos-de-Oliveira [9] showed that ArtinM induces migration of neutrophils to the peritoneal cavity or air pouch of rats and also showed that ArtinM has chemotactic activity for human neutrophils *in vitro*. The attraction occurs by haptotaxis rather than chemotaxis. The hemagglutination activity of ArtinM as well as its inhibition by carbohydrates (D-manose, D-glucose, or α-methyl-D-mannoside) suggests that ArtinM has at least two carbohydrate binding sites. One of the two binding sites may interact with carbohydrates on the neutrophil surface, and the other site binds to glycoconjugates in the extracellular matrix. The binding of ArtinM to the extracellular matrix results in a lectin gradient that directs the migration of neutrophils [42]. This ArtinM gradient can then induce the binding and the haptotactic migration of neutrophils through the blood vessels. A similar process may be occurring during ArtinM induced mast cell migration.

The interaction of mast cells with lectins has been studied *in vitro* [3], but almost nothing has been done *in vivo* due to the lack of mast cell markers. The results of this study showed that ArtinM stimulated mast cell migration and resulted in an increase in mature and immature mast cells in the bone marrow. Because the mechanisms underlying mast cell recruitment to peripheral sites are poorly understood, ArtinM may be an important tool for elucidating the molecular mechanisms involved in recruitment and migration of mast cells as well as other hematopoietic cells.
